# LOSS, SLEEP, AND HEART RATE VARIABILITY: GENDER DIFFERENCES

**DOI:** 10.1093/geroni/igz038.3338

**Published:** 2019-11-08

**Authors:** Hye Won Chai, Dylan J Jester, Soomi Lee, Susanna Joo

**Affiliations:** 1 The Pennsylvania State University, University Park, Pennsylvania, United States; 2 School of Aging Studies, University of South Florida, Tampa, Florida, United States; 3 University of South Florida, Tampa, Florida, United States; 4 Yonsei University, Seoul, Korea, Republic of

## Abstract

Death of a significant other is consistently found to have a detrimental effect on cardiovascular functioning, and such relationship may be stronger when loss is accompanied by low-quality sleep. Using data from the Biomarker project of Midlife in the United States study (n=1,310), we examined whether quality-of-sleep has an additive effect on the relationship between loss and heart rate variability (HRV). Loss was measured as losing someone close within a year of data collection, and was categorized based on the respondents’ relationship with the deceased. Relationship was categorized as: immediate family, relative, and friend. Quality-of-sleep was measured using the Pittsburgh Sleep Quality scale. Results showed that the associations among loss, sleep, and HRV differed by gender. For women, losing an immediate family was associated with worse HRV and this did not differ by quality-of-sleep. For men, death of an immediate family was associated with worse HRV only among those with poorer quality sleep. These results suggest that low-quality sleep may indicate psychophysical vulnerability for men who experienced loss, which may relate to their lower capacity for physiological adaptation.

